# Editorial: Virtual, mixed and augmented reality in cognitive neuroscience and neuropsychology, volume II

**DOI:** 10.3389/fpsyg.2024.1444412

**Published:** 2024-06-19

**Authors:** Adriana Salatino, Dalila Burin

**Affiliations:** ^1^Department of Life Sciences, Royal Military Academy, Brussels, Belgium; ^2^ADAPT Centre, School of Computer Science and Statistics, Trinity College Dublin, Dublin, Ireland

**Keywords:** virtual reality, augmented reality, cognitive neuroscience, neuropsychology, sense of agency, attention, visuospatial disorders, embodied cognition

## 1 Introduction

In recent decades, interactive and simulated experiences, such as augmented reality (AR), immersive, and semi-immersive virtual reality (VR), have garnered increasing interest in investigating cognitive functions in both healthy individuals and patients with brain injuries (Bohil et al., 2011) or learning disorders (Parsons et al., 2017). VR in particular is emerging as a new frontier in cognitive neuroscience and neuropsychological studies (Tarr and Warren, 2002), holding the potential to significantly improve our understanding of behavior and cognition, potentially revolutionizing the delivery of new therapeutic interventions. Regarding rehabilitation methods, VR can be used in different contexts, in hospitals but also at home settings to promote the development of telemedicine and self-administered therapeutic approaches (Tieri et al., 2018). In addition, VR can manipulate the environment itself (Martens et al., 2019) and/or how the agent interacts with the virtual environment (Bouzbib et al., 2023), offering a unique opportunity to develop a fully controlled digital world, where responses and reactions are highly realistic and plausible (Slater, 2009).

## 2 Research Topic content

The present Research Topic “*Virtual, Mixed, and Augmented Reality in Cognitive Neuroscience and Neuropsychology, Volume II*” is the second volume of the homonymous previous collection (Burin et al., 2022). With this second edition, we wanted to further emphasize the potential of VR in creating innovative tools for cognitive psychology research on healthy individuals and in the treatment of patients with neurological diseases, including, among others, stroke and Parkinson's disease. The Research Topic features five articles, including pilot and preliminary studies, opinions, and reviews. Based on their contributions, the research articles were grouped into two main areas: Manipulation of the virtual environment (2 papers), and Manipulation of the task in VR (3 papers).

### 2.1 Manipulation of the virtual environment

Intuitively, the environment is one of the main factors that can be controlled in VR. Every single aspect of it can be designed, to create simulations that can perfectly replicate real-life situations without losing scientific rigor. Behavioral responses and reactions in these virtual environments match those in the physical world, hence motor and cognitive tasks can be implemented in the simulations, providing safe and controlled environments for patients' rehabilitation. In particular, they are perfectly allowed to perform and repeat regular and everyday activities in a completely safe way. Faria et al. conducted a systematic review to examine the ecological validity of virtual environments in the current literature; they focus on the clinical applications for the rehabilitation of patients with acquired brain injuries, in relation to motor and cognitive daily activities. After selecting 70 articles, the authors found that (1) the most commonly used environments were streets, kitchens, supermarkets, shopping centers, and others, in this order; (2) most of the studies used screen-based technologies, while some others used visors for immersive VR; (3) most of the studies tested cognitive functions, attention, memory, and motor aspects, in this order. The use of certain environments in VR is mainly related to familiarity: higher levels of engagement seem to be associated with greater familiarity with the environment (O'Brien, 2007). This is the case of studies simulating supermarkets: shopping is a highly demanding activity, involving different cognitive and motor tasks, requiring a high level of independence. Performance evaluation in virtual supermarkets has successfully differentiated between healthy individuals and participants after a stroke (as in Kang et al., 2008), comparably to what happens in the physical world. Moreover, virtual supermarkets have been effectively used to train upper limb motor functions with similar effectiveness to conventional therapy (as in Demers and Levin, 2020). Curiously, another study in our Research Topic exploited the same virtual environment: De Simone et al. reproduced a fully immersive supermarket where patients with Parkinson's had to complete a planning task (planning a route to pick certain products), a shifting task (collecting certain items alternating categories) and an updating task (remembering the number of products passing by the cashier). Participants were evaluated for cognition, memory, psychiatric symptoms, autonomy, and quality of life before, immediately after, and 2 months after the 4-week intervention period, where they repeated the same tasks at different levels of difficulty. As of today, the data collection is still ongoing, but the prediction is that the cognitive functions of Parkinson's patients, especially those showing signs of mild cognitive impairment, will improve. To summarize, the reviewed studies by Faria et al. and the preliminary data offered by De Simone et al. demonstrate that this technological approach is more engaging than traditional approaches, allows for more intensive training, provides immediate feedback, and the tasks in the virtual environment have greater authenticity and validity.

### 2.2 Manipulation of the tasks in VR for rehabilitation

After the environment itself, the following level of manipulation concerns the agent's interaction with the virtual environment, especially in terms of tasks. VR provides a controlled, immersive, and adaptable environment for the execution of motor and cognitive tasks, that can be tailored to meet specific needs, which makes it the ideal setting for rehabilitation. In their opinion article included in our Research Topic, Zhang et al. focus on rehabilitative tasks in VR for cognitive impairment in post-stroke patients: they support the conclusions that VR technology can enhance cognitive functions (especially executive functions, as in Rogers et al., 2023) and boost engagement (as in Faria et al., 2016); furthermore, VR offers multisensory stimulation, creating a controlled but realistic experience that benefits both somatic and cognitive functions in post-stroke patients. The authors confirm that the use of VR in stroke rehabilitation is expected to increase, showing significant potential for the treatment of post-stroke patients. While VR technology might be complicated to tolerate by the elderly (discomfort, dizziness, fatigue), it has only recently been used in pediatric rehabilitation. Di Giusto et al. tested a semi-immersive VR rehabilitation program for children with specific learning disorders (dyslexia, dysorthography, dysgraphia, dyscalculia, developmental coordination disorder): 24 children aged 7–11 years performed two games including tasks for visual attention, working memory, inhibitory control and shifting, as well as the upper limb control movements, for 6 weeks (twice a week, 45 min each). Their visual attention, inhibition, and planning skills were assessed before, immediately after, and 6 months after the end of the program. Preliminary results showed that after the 6-week rehabilitation program, the scores for visual attention, inhibition, flexibility, and planning abilities in children with learning disorders were significantly higher than before the intervention, and these improvements were maintained after six months. The authors argue that children with learning disorders feel free to interact in the VR environment, avoiding stressful and demanding academic tasks, and also receive appropriate feedback. One limitation of this study is the lack of a control group. VR tasks can be used not only for the rehabilitation of specific disorders but also for their assessment. Uimonen et al. developed a novel immersive VR task battery, including eye tracking too, to assess visual unilateral neglect in patients in acute state post-stroke. The VR task battery included an extinction task (reporting the position of a target), a storage task (locating a specific target), and a shooting task (choosing targets by staring at them). Preliminary results showed that the VR task battery differentiated patients with neglect from control subjects: in the extinction task, patients detected fewer bilateral (simultaneously presented) targets on the left side than controls (stroke patients without neglect), and in the storage task, patients' gaze was less focused on the left side than the control subjects, consistently with previous studies. The authors argue that the combination of VR with gaze-based responses excluded the possible distorting effect of motor action on task performance. VR-based technology is demonstrated to be user-friendly for post-stroke patients, as supported by Zhang et al.. In summary, VR allows for the creation of engaging and motivating therapeutic activities that can enhance patient participation and adherence to rehabilitation programs. It provides real-time feedback and can simulate a wide range of scenarios, enabling various clinical populations (elderly and children) to practice functional tasks and movements safely. Furthermore, VR can be used to detect and assess specific disorders, ultimately leading to more effective and personalized rehabilitation outcomes.

## 3 Outlook

In this Research Topic, VR is described in its many facets as an extraordinary tool for controlling the environment and interacting with agents. Its effectiveness is demonstrated through applications on different clinical populations (children with learning disorders, post-stroke elderly), with various tasks and batteries of tasks, and different environments simulating real-life scenarios. Executive functions seem to be the most appropriate to be explored, tested, and restored in VR. The preliminary evidence presented in this collection of papers emphasizes the increasing trend toward the use of VR in neuropsychology and cognitive neuroscience.

## Author contributions

AS: Writing – original draft, Writing – review & editing. DB: Writing – original draft, Writing – review & editing.

## References

[B1] Bohil C. J. Alicea B. Biocca F. A. (2011). Virtual reality in neuroscience research and therapy. Nat. Rev. Neurosci. 12, 752–762. 10.1038/nrn3122 22048061

[B2] Bouzbib E. Pacchierotti C. Lecuyer A. (2023). When tangibles become deformable: Studying pseudo-stiffness perceptual thresholds in a VR grasping task. IEEE Trans. Vis. Comput. Graph. 29, 2743–2752. 10.1109/TVCG.2023.3247083 37028356

[B3] Burin D. Salatino A. Ziat M. (2022). Editorial: Virtual, mixed, and augmented reality in cognitive neuroscience and neuropsychology. Front. Psychol. 13:1010852. 10.3389/fpsyg.2022.1010852 36118497 PMC9479729

[B4] Demers M. Levin M. F. (2020). Kinematic validity of reaching in a 2D virtual environment for arm rehabilitation after stroke. IEEE Trans. Neural Syst. Rehabil. Eng. 28, 679–686. 10.1109/TNSRE.2020.2971862 32031942

[B5] Faria A. L. Andrade A. Soares L. (2016). Benefits of virtual reality based cognitive rehabilitation through simulated activities of daily living: a randomized controlled trial with stroke patients. J. Neuro. Eng., 13:96. 10.1186/s12984-016-0204-z 27806718 PMC5094135

[B6] Kang Y. J. Ku J. Han K. Kim S. I. Yu T. W. Lee J. H.. (2008). Development and clinical trial of virtual reality-based cognitive assessment in people with stroke: preliminary study. Cyber Psychol. Behav. 11, 329–339. 10.1089/cpb.2007.0116 18537503

[B7] Martens M. A. Antley A. Freeman D. Slater M. Harrison P. J. Tunbridge E. M. (2019). It feels real: physiological responses to a stressful virtual reality environment and its impact on working memory. J. Psychopharmacol. 33, 1264–1273. 10.1177/0269881119860156 31294651 PMC6764008

[B8] O'Brien J. (2007). Simulating the homes of stroke patients: can virtual environments help to promote engagement in therapy activities? Virtual Rehabil. 2007, 23–28. 10.1109/ICVR.2007.4362124

[B9] Parsons T. D. Riva G. Parsons S. Mantovani F. Newbutt N. Lin L.. (2017). Virtual reality in pediatric psychology. Pediatrics 140, S86–S91. 10.1542/peds.2016-1758I 29093039

[B10] Rogers J. M. Duckworth J. Middleton S. Steenbergen B. (2023). Elements virtual rehabilitation improves motor, cognitive, and functional outcomes in adult stroke: evidence from a randomized controlled pilot study. J. Neuro. Eng. 16:56. 10.1186/s12984-019-0531-y 31092252 PMC6518680

[B11] Slater M. (2009). Place illusion and plausibility can lead to realistic behaviour in immersive virtual environments. *Philos. Trans. R. Soc. Lond. B*,. Biol. Sci. 364, 3549–3557. 10.1098/rstb.2009.0138 19884149 PMC2781884

[B12] Tarr M. J. Warren W. H. (2002). Virtual reality in behavioral neuroscience and beyond. Nat. Neurosci. 5, 1089–1092. 10.1038/nn948 12403993

[B13] Tieri G. Morone G. Paolucci S. Iosa M. (2018). Virtual reality in cognitive and motor rehabilitation: facts, fiction and fallacies. Expert Rev. Med. Devices 15, 107–117. 10.1080/17434440.2018.1425613 29313388

